# Imaging evaluation of nonobstetric conditions during pregnancy: what every radiologist should know

**DOI:** 10.1590/0100-3984.2019.0059

**Published:** 2020

**Authors:** Ana Paula Campos Rocha, Rafael Lourenço Carmo, Rodolfo Ferreira Queiroz Melo, Daniel Nogueira Vilela, Orlando Silqueira Leles-Filho, Luciana Costa-Silva

**Affiliations:** 1 Instituto Hermes Pardini, Belo Horizonte, MG, Brazil.; 2 Beneficência Portuguesa de São Paulo, São Paulo, SP, Brazil.; 3 Hospital das Clínicas da Universidade Federal de Minas Gerais (UFMG), Belo Horizonte, MG, Brazil.; 4 Universidade Estadual de Montes Claros (Unimontes), Montes Claros, MG, Brazil.

**Keywords:** Pregnancy/radiation effects, Fetus/radiation effects, Emergencies, Radiography/adverse effects, Tomography, X-ray computed/adverse effects, Radiation protection, Gravidez/efeitos da radiação, Feto/efeitos da radiação, Emergências, Radiografia/efeitos adversos, Tomografia computadorizada/efeitos adversos, Proteção radiológica

## Abstract

In recent decades, there has been a significant increase in the number of imaging examinations performed on pregnant patients. That increase has occurred across the various modalities, including ultrasound, computed tomography, and magnetic resonance imaging. However, little is known about the risks that these examinations generate for the mother and fetus, related to the use of ionizing radiation or the use of contrast media. When pregnant patients are submitted to imaging studies, the principles of protection established by the International Commission on Radiological Protection should always be respected, to avoid injury to the pregnant woman and the fetus. The potential deleterious effects on the fetus must be weighed against the damage caused by not performing an examination that is clearly indicated, given that a delayed or missed diagnosis can be even more harmful to the health of the mother and of the fetus itself. The purpose of this review article is to address concerns regarding the safety of imaging methods used during pregnancy, as well as to identify typical clinical situations that require decisions to be made about the indication and optimal planning of imaging examinations.

## Abstract (pt)

Nas últimas décadas observou-se um aumento expressivo no número de exames de imagem realizados em pacientes gestantes. Este aumento inclui diferentes modalidades, como a ultrassonografia, a tomografia computadorizada e a ressonância magnética. Entretanto, ainda se sabe pouco sobre os riscos que esses exames geram para o binômio mãe-feto, seja no contexto da utilização de radiação ionizante ou da utilização de diferentes meios de contraste. Ao se submeter pacientes grávidas a estudos por imagem, deve-se sempre respeitar os princípios de limitação do uso estabelecidos pela Comissão Internacional de Proteção Radiológica, visando a poupar a gestante e o feto de danos evitáveis. Ressalta-se que os potenciais efeitos deletérios sobre o feto se contrapõem aos danos causados pela não realização de um exame bem indicado, já que um diagnóstico protelado ou perdido pode ser ainda mais nocivo para a saúde materna e do próprio concepto. O objetivo deste artigo de revisão é esclarecer dúvidas sobre a segurança dos métodos de imagem durante o período gestacional, bem como identificar situações clínicas típicas que exigem decisões sobre a indicação e o planejamento otimizado dos exames de imagem.

## INTRODUCTION

The frequency of imaging examinations during pregnancy has increased in recent decades, and, although ultrasound is the main method used, computed tomography (CT) and (especially) magnetic resonance imaging (MRI) are often necessary in order to clarify the diagnosis^(1-3)^. In this context, questions have been raised regarding the potential harm to the health of the embryo and the fetus, notably in relation to the use of ionizing radiation and contrast media. However, the potential deleterious effects on the fetus must be weighed against the damage caused by not performing an examination that is clearly indicated, given that a delayed or missed diagnosis can be even more harmful to the health of the mother and fetus. Thus, it is important to know the main imaging methods suitable for each clinical situation, allowing the diagnostic value and the potential harmful effects of each situation to be estimated, justifying their use for the benefit of the patient.

## IMAGING METHODS

Imaging methods that do not use ionizing radiation are preferred during pregnancy. Ultrasound and MRI have advantages over CT. However, CT can provide important data, contributing to diagnosis in various situations.

To reduce the risks to which the pregnant woman and the fetus will be exposed, it is important to remember the fundamental principles of radiation protection. Chief among these is the principle of dose limitation, which states that the lowest possible dose needed in order to obtain the diagnosis should be used, a principle that the International Commission on Radiological Protection has named the “as low as reasonably achievable” (ALARA) principle^(4)^.

A legal aspect to be considered by obstetricians and radiologists before conducting imaging studies is use of an informed consent form. Obtaining informed consent from pregnant women is considered good medical practice, in order to document patient understanding about alternative diagnoses, as well as about the harms and benefits related to the examination to be conducted. Informed consent should be obtained from all pregnant women undergoing cross-sectional examinations, including MRI and CT^(5)^.

### Ultrasound

Ultrasound is a widely available, low-cost imaging method and the examination of choice in most clinical situations during pregnancy, which also presents, as an advantage, the fact that it does not expose patients to ionizing radiation or contrast media. There have been no reports of biological effects (on the embryo or fetus) related to the use of this method^(6,7)^. Noteworthy disadvantages of ultrasound are that it is operator-dependent, shows reduced accuracy in some clinical situation, low resolution and less penetration in abdominal pregnancy^(5)^.

### Conventional radiography and CT

Conventional radiography and CT are relevant in the diagnosis of many diseases. The former is a widely available, low-cost method and exposes the patient to relatively low doses of radiation. However, CT, despite its lower availability and higher dose of radiation, eliminates the disadvantage of overlapping structures, allowing greater diagnostic accuracy in most clinical situations.

There have been no well-controlled studies in pregnant women about the risks of ionizing radiation used by these imaging methods. Most of the information currently available is based on case reports and the extrapolation of data from research on the survivors of nuclear explosions in Japan and the Chernobyl accident^(6)^.

For a pregnant woman, the biological effects of radiation are the same as those suffered by a woman who is not pregnant. However, undesirable biological effects on the conceptus include intrauterine death and organic defects (teratogenic effect), as well as altered formation and migration of the central nervous system, resulting in varied degrees of cognitive defect, depending on the gestational age at exposure^(8)^.

The biological effects of radiation are classified as deterministic or stochastic. Deterministic effects are related to exposure to high doses of radiation and depend directly on the dose delivered, such as spontaneous abortion, possible defects, and other teratogenic effects on or cognitive defects in the conceptus. Stochastic (i.e., random) effects are not related to the direct, immediate effect of radiation and may occur months or years after exposure. The stochastic effects are not related to exposure to a specific threshold dose of radiation. However, the probability of occurrence is proportional to the dose, and the most relevant biological manifestations are mutations and carcinogenesis^(9)^.

The occurrence of the biological effects depends on the dose of radiation absorbed and is further related to the gestational age of the fetus or embryo. In general, the absorption of low doses of radiation may cause temporary cell damage that can be repaired by the body. However, high doses of radiation can disrupt cell development and maturation, leading to fetal death or malformations^(9,10)^.

### Fetal development

In general, radiation doses lower than 50 mGy are not related to fetal abnormalities or spontaneous abortion^(8,11)^. As illustrated in Table 1, the fetus is not exposed to doses approaching this threshold in most of the imaging examinations used in clinical practice, as long as they are performed with protocols optimized for diagnosis using the lowest dose of radiation required (i.e., in accordance with the ALARA principle).

**Table 1:** Estimated fetal doses in conventional radiography and CT examinations.

Examination	Estimated fetal dose (mGy)
Conventional radiography	
Cervical spine (anteroposterior, lateral), extremities, thorax (posteroanterior, lateral), thoracic spine	< 0.003
Lumbar spine (anteroposterior, lateral)	1
Abdomen (anteroposterior)	< 3
CT	
Head	0
Thorax (routine), thorax (CT angiography for suspected PTE)	0.2
Abdomen	4
Abdomen and pelvis	25
CT angiography of the aorta	34

### Biological effects of ionizing radiation and gestational age

The embryo is more sensitive to the effects of ionizing radiation in the first two weeks of the embryonic stage of development (equivalent to a gestational age of 3-4 weeks), the time at which the exposed conceptus will remain intact or will be reabsorbed or aborted^(6,8)^. During that period, exposure to a dose greater than 100 mGy is considered life-threatening^(8)^.

From week 3 to week 15 of the embryonic stage of development (gestational age of 5-17 weeks), embryogenesis occurs, and damage to the embryo, as well as disorders in cell proliferation and migration, may arise from radiation-induced cell death. In that stage, severe malformations can incur, including those affecting the developing central nervous system. When the fetus is exposed to doses higher than 100 mGy, there can even be cognitive defects and a reduced intelligence quotient. The frequency and severity of these risks increase in direct proportion to the dose of radiation^(8)^. We emphasize, however, that it is unlikely that the fetus will be exposed to such high doses in routine diagnostic examinations, which are performed with the radiation field directly above the uterus. Between weeks 18 and 27 of the gestational stage of development (corresponding to a embryonic age of 16-25 weeks), no intelligence quotient deficits have been detected at any diagnostic dose. After week 27 (embryonic age of 25 weeks), there are significant risks to the fetus in relation to deterministic effects^(8)^, as can be seen in Table 2.

**Table 2:** Summary of potential deterministic effects on the fetus associated with exposure to ionizing radiation.

Period (age)			Dose / deterministic effects		
Menstrual/gestational	Embryonic		< 50 mGy	50-100 mGy	> 100 mGy
0-2 weeks	Prior to conception		None	None	None
3-4 weeks	1-2 weeks		None	Probably none	Possible spontaneous abortion
5-10 weeks	3-8 weeks		None	Potential effects uncertain and too subtle to be clinically detectable	Possible malformations, increasing in probability as doses increase
11-17 weeks	9-15 weeks		None	Potential effects uncertain and too subtle to be clinically detectable	Risk of cognitive deficits becoming more frequent and more severe as doses increase
18-27 weeks	16-25 weeks		None	None	Deficits in intelligence not detectable at diagnostic doses
> 27 weeks	> 25 weeks		None	None	None are applicable to diagnostic medicine

*Note:* Although there are no consistent data in the literature, stochastic effects are suspected, there being a potential risk of developing cancer, especially leukemia, in childhood.

### Iodinated contrast media

There have been few studies on the use of intravenous iodinated contrast in pregnant women, and its effects on human fetuses are still not completely understood^(12)^. It has been demonstrated that iodinated contrast media cross the placenta in measurable quantities, although tests on animals have shown no deleterious effects. There have been no well-controlled studies in humans. However, there are also no documented cases of potential damage to human embryos or fetuses arising from maternal intravenous use of iodinated contrast media^(12)^. Therefore, the American College of Radiology does not recommend avoiding the use of intravenous iodinated contrast in pregnant or potentially pregnant patients when necessary for diagnosis. The US Food and Drug Administration has supported that position by classifying most iodinated contrast media as pregnancy risk category B drugs.

### MRI

The main advantages of MRI are the lack of exposure to ionizing radiation, its capacity to obtain multiplanar images, and its excellent resolution in the evaluation of soft tissues^(5)^. Secondary to the electromagnetic field itself, the potential risks to the conceptus are tissue heating caused by high-frequency pulses, hearing damage caused by high-frequency noise, and cell migration defects during the first trimester^(2)^. Despite these theoretical concerns, there have been no reports of adverse effects in pregnant women or fetuses who underwent MRI^(13)^. In a study conducted in Canada and involving 1,737 pregnant women undergoing MRI in the first trimester, Ray et al.^(13)^ followed the offspring until the age of four years and found no statistically significant increase in the risk of stillbirth or neonatal death, congenital abnormalities, neoplasms, and vision or hearing loss.

The use of MRI in 1.5-T scanners is considered safe, whereas examinations in 3.0-T scanners are discouraged, due to the greater potential of tissue heating and the lack of adequate studies to date^(2,6)^. The use of MRI is recommended during any stage of pregnancy, when other methods that do not use ionizing radiation have not clarified the clinical situation, provided that the examination is relevant to define the diagnosis and treatment of the pregnant patient and/or the fetus and that it is not prudent to wait until the patient is no longer pregnant^(14)^.

### Paramagnetic contrast media

In relation to gadolinium, there have been no reports of adverse mutagenic effects in human fetuses at the doses regularly used. However, there have been no well-controlled studies about the teratogenic effects of this contrast medium in human conceptuses. Likewise, there have been no reported cases of nephrogenic systemic fibrosis triggered by the use of gadolinium during pregnancy, although there is a potential risk to the mother and to the child^(12)^.

In the study cited above, conducted in Canada, Ray et al.^(13)^ found no increased risk of congenital anomalies among the fetuses of patients who underwent gadolinium-enhanced MRI during pregnancy, in comparison with those of patients who did not. However, the authors reported a statistically significant increase in the risk of intrauterine death and neonatal death, as well as of some rheumatological, inflammatory or infiltrative conditions, especially those related to the use of gadolinium in the first trimester^(13)^. It should be emphasized that theirs was a single, retrospective study with methodological limitations and that there are no other robust studies available in the literature^(12)^.

Due to the uncertain effects on children who had intrauterine contact with paramagnetic contrast media, drugs such as gadolinium should be used with caution during pregnancy. The American College of Radiology recommends that gadolinium be used only if the benefit outweighs the potential risk to the fetus and that it be administered at the lowest doses possible for diagnosis^(12)^.

## MAIN APPLICATIONS OF IMAGING EXAMINATIONS

### Evaluation of the pregnant patient with abdominal pain

The choice of imaging method to be used in the abdominal evaluation of the pregnant patient is complex and should take into account the urgency in diagnostic confirmation, the main clinical hypotheses, the results of previous examinations, and the risks that these examinations generate for the mother and fetus. The major nonobstetric clinical entities in need of an imaging evaluation of the abdomen are those that require urgent attention, such as acute abdomen (particularly appendicitis and biliary tract diseases) and obstructions of the urinary tract.

Below is a brief discussion on some of the major clinical conditions, with recommendations on the imaging methods of choice for investigating each condition. At the end of each discussion are algorithms with suggested imaging protocols for the evaluation of the major nonobstetric conditions that occur during pregnancy.

#### Acute appendicitis

Acute appendicitis is the leading cause of acute surgical abdomen in pregnant women, with an estimated prevalence of 50-70 cases/1,000 patients^(5,7)^. Appendicitis in pregnant patients increases the risk of premature labor, is associated with higher rates of fetal morbidity, and demonstrates a higher risk of perforation, in comparison with appendicitis in nonpregnant patients^(7)^. In addition to the greater potential severity, the clinical diagnosis is hampered, given that several of the clinical alterations caused by the disease (nausea, vomiting, and leukocytosis) are common during pregnancy^(7)^.

The sensitivity of ultrasound in the detection of acute appendicitis reported in the literature is variable. That is probably due to the fact that ultrasound is an operator-dependent method. The specificity, however, is high, at around 95%^(5,7)^. Therefore, ultrasound is the modality of choice for initial evaluation, because it does not expose the fetus to radiation, as well as because it can be used in evaluating the abdomen and the pelvis in search of alternative diagnoses, MRI and CT therefore being second-line methods.

**Figure 1:**
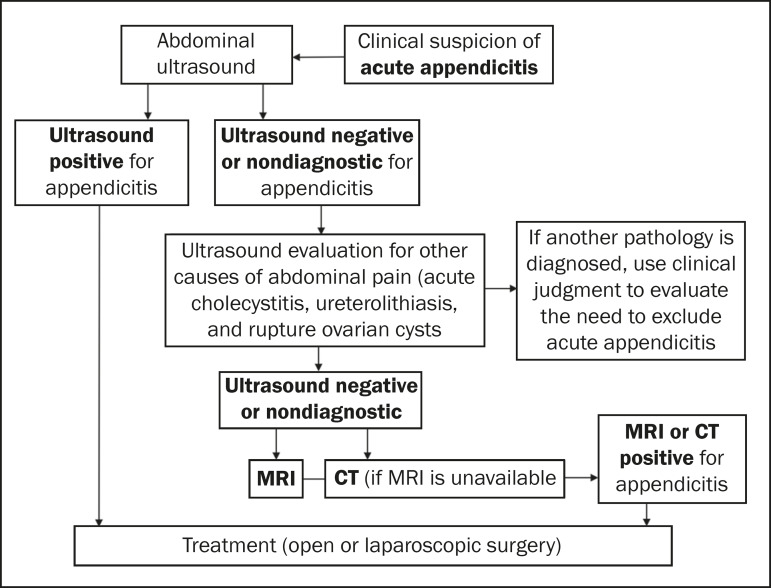
Algorithm for the imaging evaluation of suspected appendicitis in pregnant patients.

It should be borne in mind that, in the final phases of pregnancy, the appendix is displaced by the gravid uterus, promoting higher migration and rotation of this structure, reducing the sensitivity of an ultrasound examination. Nevertheless, most authors recommend the use of ultrasound as the first-line imaging study^(5,7)^. Recent studies have shown MRI to be highly accurate, allowing it to be considered the main alternative diagnostic tool^(5,7)^. For the detection of acute appendicitis, CT also shows high accuracy, with the advantages of greater availability, compared with MRI, and less dependence on the examiner. However, due to the risk of fetal exposure to radiation, it should be considered a secondary method, with indications restricted to cases that could not be resolved with ultrasound or MRI^(5,7)^ (Figure 1).

#### Biliary tract diseases

Although pathological involvement of the gallbladder and biliary tract is uncommon during pregnancy, acute cholecystitis is still the second leading cause of nonobstetric surgical intervention in pregnant women, occurring in 1 of every 1,600-10,000 pregnancies. Other important conditions in this context are obstructive choledocholithiasis and biliary pancreatitis^(7,15)^.

**Figure 2:**
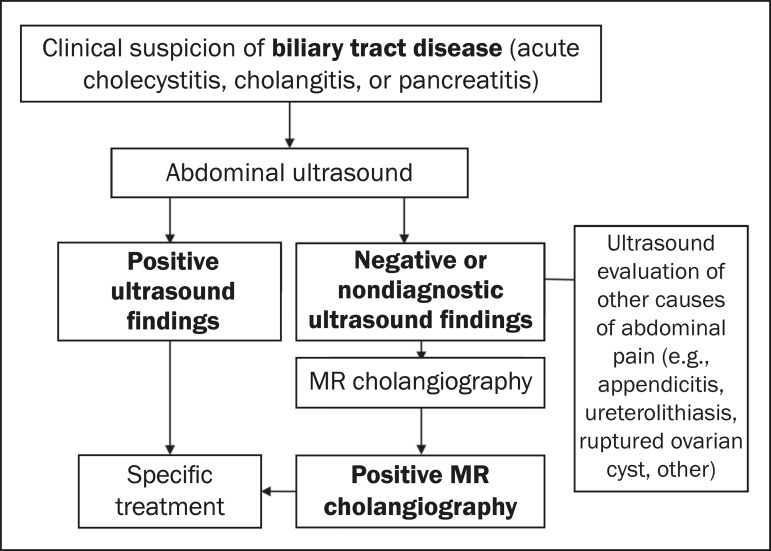
Algorithm for the imaging evaluation of suspected biliary tract disease in pregnant patients.

**Figure 3:**
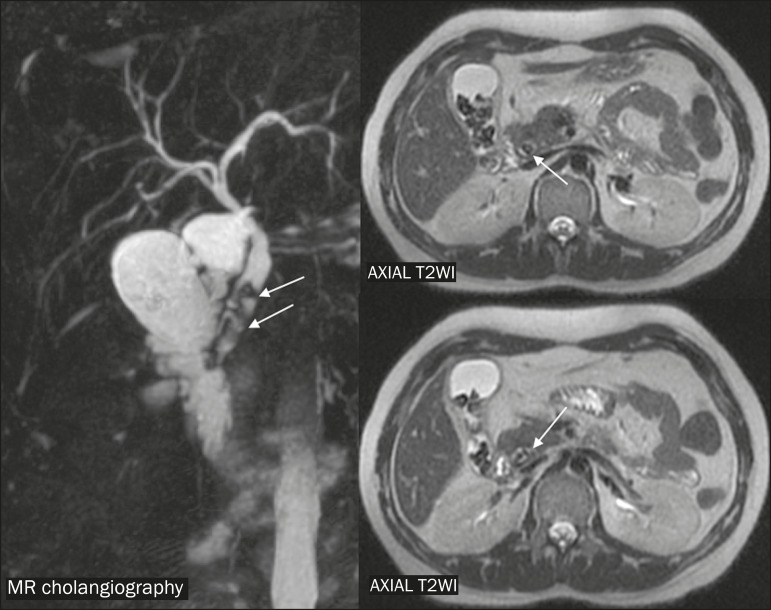
Magnetic resonance cholangiography and axial T2-weighted image (axial T2WI) from an MRI scan, showing choledocholithiasis, highlighting impacted calculi in the distal common bile duct (arrows), with mild dilatation of the upstream biliary tract.

For the etiological clarification of biliary tract diseases, MRI shows high sensitivity and specificity (98% and 84%, respectively), although ultrasound is still the first-line examination in cases of suspected acute biliary complications in pregnant women, because of its wide availability and low cost^(5,7)^. Therefore, MRI continues to be an alternative for cases in which the ultrasound findings are inconclusive (Figures 2 and 3).

#### Ureterolithiasis

Urinary tract obstruction caused by ureterolithiasis is another potential cause of abdominal pain in pregnancy. Potential complications of this condition include pyelonephritis and sepsis, as well as premature labor induced by a kidney stone, with or without concomitant infection^(15)^.

**Figure 4:**
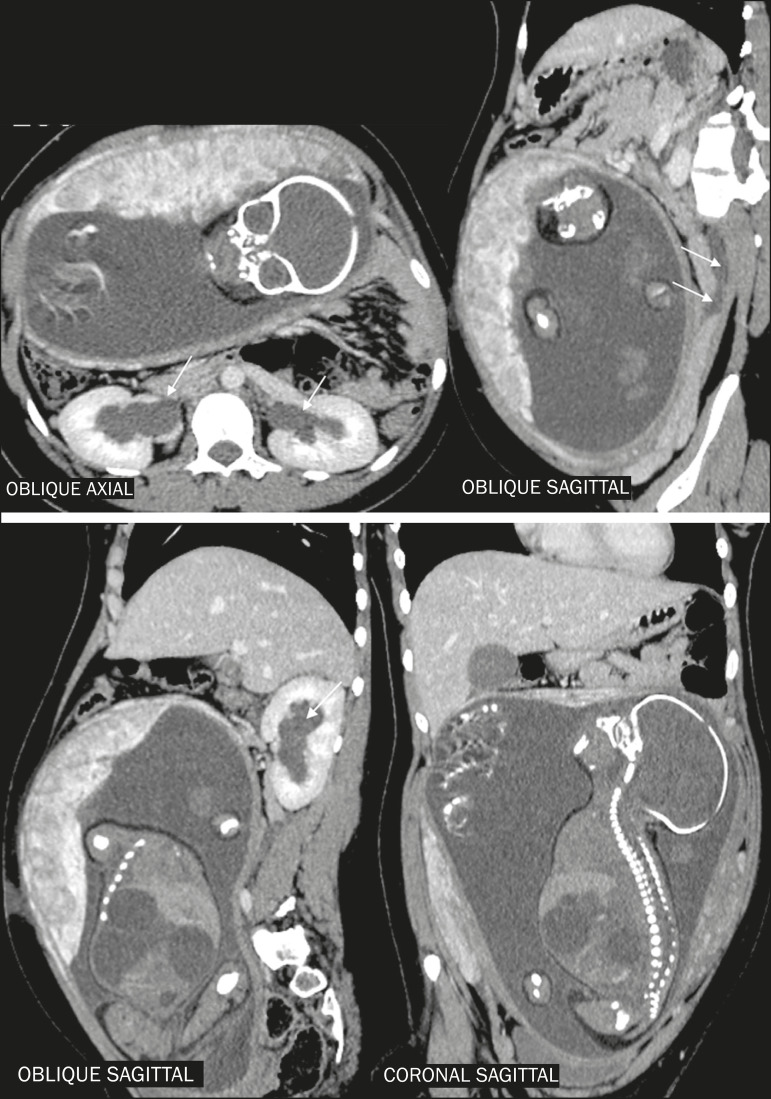
Reconstructions of contrast-enhanced CT scans in the oblique axial, sagittal, and coronal planes showing a patient with physiological hydronephrosis in pregnancy. Note the mild bilateral dilation of the renal pelvis and extrinsic compression of the left ureter by the gravid uterus (arrows). Another incidental finding was dilation of the bowel loops of the fetus, together with polyhydramnios, indicative of intestinal obstruction.

**Figure 5:**
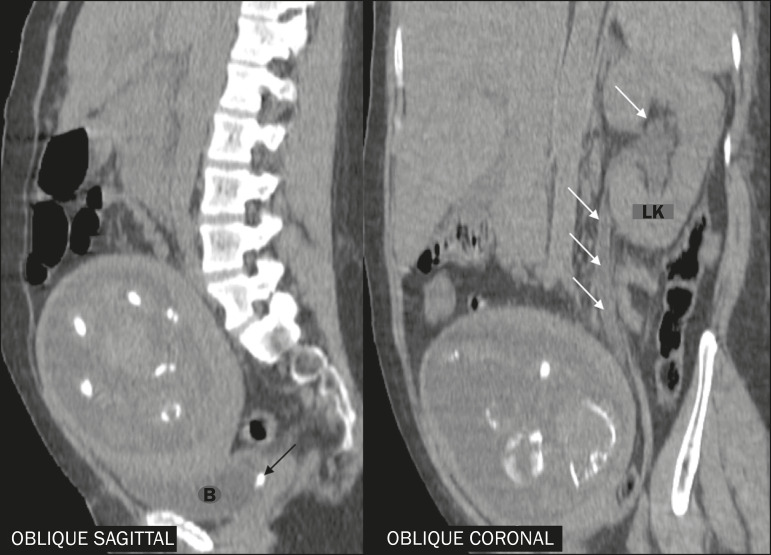
Reconstructions of unenhanced CT scans in the oblique axial, sagittal, and coronal planes, showing ureterolithiasis in a pregnant patient. Note the impacted gallstone in the left distal ureter (black arrow) and the ipsilateral upstream hydronephrosis (arrows). B, bladder; LK, left kidney.

During pregnancy, there may be physiological dilatation of the collecting system (hydroureteronephrosis), conditioned by ureteral relaxation related to hormonal changes and extrinsic compression of the ureter by the gravid uterus, more commonly on the right side (Figure 4). Therefore, the main challenge of diagnostic imaging in these cases is to differentiate physiological hydronephrosis from obstructive hydronephrosis, usually secondary to ureterolithiasis^(5,7,15)^, as depicted in Figure 5.

Despite the low sensitivity of ultrasound for identifying ureteral calculi in pregnant women, it is the first examination performed when there is suspicion of obstructive ureterolithiasis, because it is widely available, has a favorable cost-benefit ratio, and permits the evaluation of hydroureteronephrosis, with the possibility of diagnosing ureterolithiasis without the use of ionizing radiation^(5,7,15)^. If the ultrasound findings are inconclusive, MRI is still the first alternative. That is because MRI has high accuracy for detecting dilation in the urinary collection system and the point of obstruction and, despite frequently not making the stone visible, shows indirect findings that afford a differentiation between physiological dilation and dilation of obstructive origin, such as renal enlargement, the presence of perirenal fluid, and an abrupt change in ureteral caliber above or below the uterus, which can be identified in obstructive dilations^(7,15)^.

**Figure 6:**
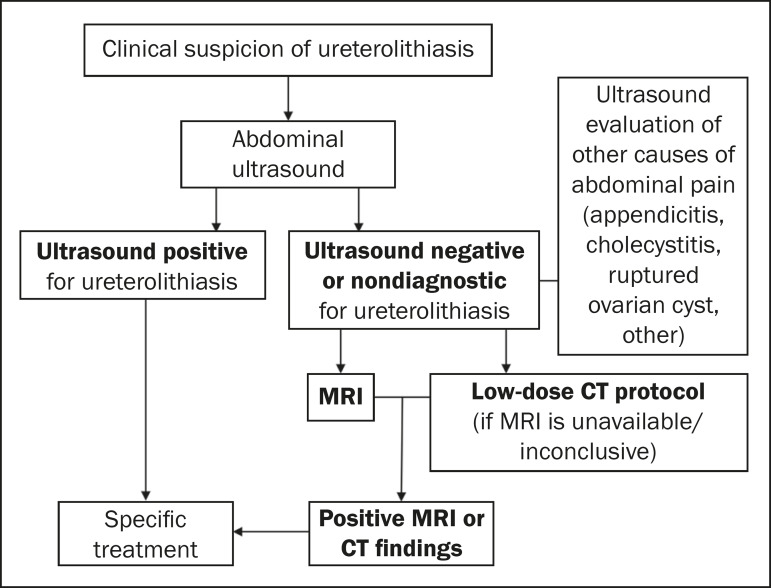
Algorithm for imaging evaluation of pregnant women suspected of having ureterolithiasis.

If any diagnostic uncertainty remains after ultrasound and MRI have been performed, CT is considered the definitive diagnostic method. Because the embryo or fetus comes into the field of view of an abdominal or pelvic scan, special care must be taken to ensure the safety of the conceptus. Standard CT protocols expose the fetus to a dose of radiation estimated at 25 mGy (Table 1). Low-dose protocols for specific investigation of kidney stones (Figure 6), using low tube current and voltage (160 mA and 140 kVp, respectively) in a multidetector scanner with at least 16 channels, specify a fetal radiation dose limit of 11.7 mGy and should be preferred over standard protocols^(7)^.

Other urological conditions, such as acute pyelonephritis, should be borne in mind as differential diagnoses. In this context, ultrasound and MRI should be used, when possible, as screening methods and methods that provide greater anatomical detail.

### Evaluation of the pregnant patient with dyspnea

The main causes of dyspnea during the gestational period include pulmonary thromboembolism (PTE), community-acquired pneumonia, pulmonary edema, asthma exacerbation, and amniotic fluid aspiration. Chief among those is PTE, which is a major cause of maternal mortality and is diagnosed by imaging.

In pregnant women, an increase in venous stasis and changes in coagulation factors (state of hypercoagulability) result in a five times greater risk of deep vein thrombosis (DVT). Other predisposing factors include obesity, advanced maternal age, thrombophilia, antiphospholipid antibody syndrome, trauma, surgery, and immobility^(16)^.

The main clinical symptoms of acute PTE include dyspnea, pleuritic chest pain, coughing, tachypnea, tachycardia, hypoxemia, pain, and asymmetric edema in the lower extremities^(16)^. When there is suspicion of PTE, diagnostic confirmation by imaging examination is required.

The examination of choice for the investigation of PTE is compression ultrasound, which does not involve the use of contrast agents or ionizing radiation^(5)^. If DVT is found, treatment should be instituted and additional examinations are not required. If compression ultrasound is unavailable, the ultrasound results are negative, or there are no coexisting symptoms of DVT, CT angiography of the pulmonary arteries or ventilation/perfusion scintigraphy is recommended, given that the final diagnosis of this pathology is accomplished by showing the high probability for PTE in ventilation/perfusion scintigraphy or by direct visualization of an arterial thrombus on CT angiography^(16,17)^.

Although the choice between CT angiography and ventilation/perfusion scintigraphy is challenging and there are conflicting data in the literature, the diagnosis and treatment of PTE should not be delayed because the preferred imaging method is not available. Therefore, the choice should be based primarily on which method is available, as well as on clinical judgment, patient preferences, and the presence of comorbidities-scintigraphy is preferred in patients with nephropathy who are allergic to iodinated contrast, whereas CT angiography is preferred in patients with lung disease^(17)^. Scintigraphy has the advantage of lower maternal exposure to radiation, whereas CT angiography enables the identification of alternative diagnoses^(5,17)^.

**Figure 7:**
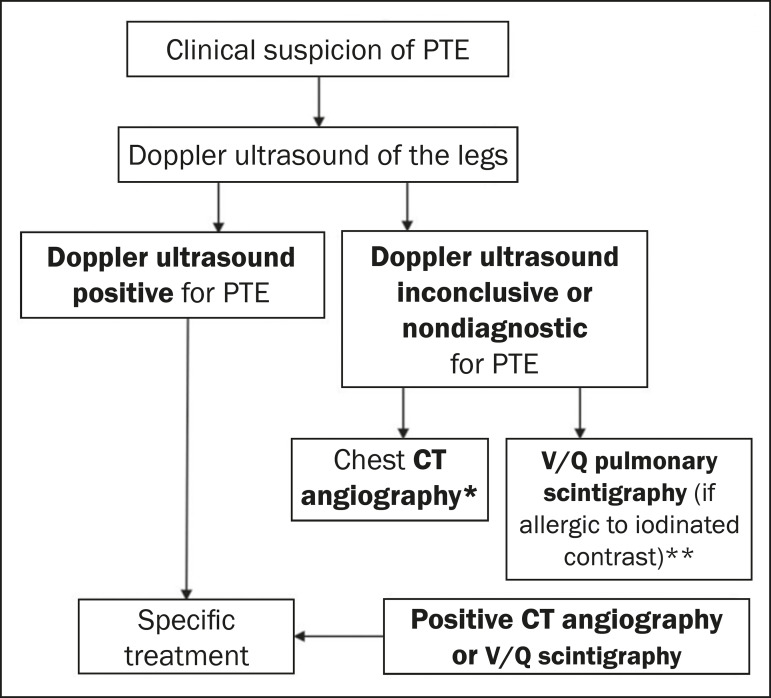
Algorithm for the imaging evaluation of suspected PTE in pregnant patients. V/Q, ventilation/perfusion. * If CT angiography is considered technically inadequate, Doppler ultrasound of the legs should be performed or CT angiography should be repeated. ** If the V/Q scintigraphy is nondiagnostic, CT angiography should be performed.

It is currently recommended that, if examinations other than compression ultrasound are required, chest X-ray should initially be performed. If the X-ray shows no changes, ventilation/perfusion scintigraphy should be performed (Figure 7). Otherwise, CT angiography is preferable and is also useful in cases in which the scintigraphy findings are inconclusive and the level of clinical suspicion remains high^(17-19)^.

Pregnant patients may show cardiogenic or noncardiogenic pulmonary edema. In the latter case, the main predisposing factors are the following: the use of tocolytics; pre-eclampsia or severe eclampsia; and iatrogenic administration of large quantities of fluids. The diagnosis is usually made by means of anamnesis and physical examination. A chest X-ray can show signs of pulmonary venous congestion-consolidations, central “butterfly wing” opacities, interlobular septal thickening (Kerley B lines)-, and CT is not required in most cases^(16)^.

Community-acquired pneumonia is a relatively common cause of respiratory failure in pregnant women, in which clinical findings include coughing with purulent expectoration, fever, dyspnea, tachycardia, and tachypnea. Some patients also develop hypoxemia, pleuritic pain, gastrointestinal symptoms, and mental confusion. The diagnosis is made on the basis of radiologic evidence of focal or multifocal airspace disease (consolidations or opacities with ground-glass attenuation), as well as a clinical history consistent with the disease and bacteriological confirmation. In relation to imaging methods, chest X-ray is the examination of choice and is sufficient to confirm the diagnosis in most cases.

Other causes of dyspnea in pregnant patients include asthma attacks and amniotic fluid embolism. Those conditions are diagnosed primarily on the basis of the clinical findings, which makes the use of imaging methods unnecessary.

### Evaluation of the polytraumatized pregnant patient

Automobile accidents and domestic violence are the main causes of trauma, often high-energy trauma, during pregnancy. In such cases, the evaluation of pregnant patients becomes challenging, given that the presence of the fetus implies the evaluation of two patients at risk^(11,20)^.

Initially, maternal survival should be prioritized, with procedures centered on the hemodynamic stability of the pregnant patient. In this context, additional tests that are required should not be delayed, given that insufficient diagnosis can lead to maternal and fetal death^(5)^. Thus, the benefits of imaging examinations outweigh the potential risks, even when using ionizing radiation^(20)^.

**Figure 8:**
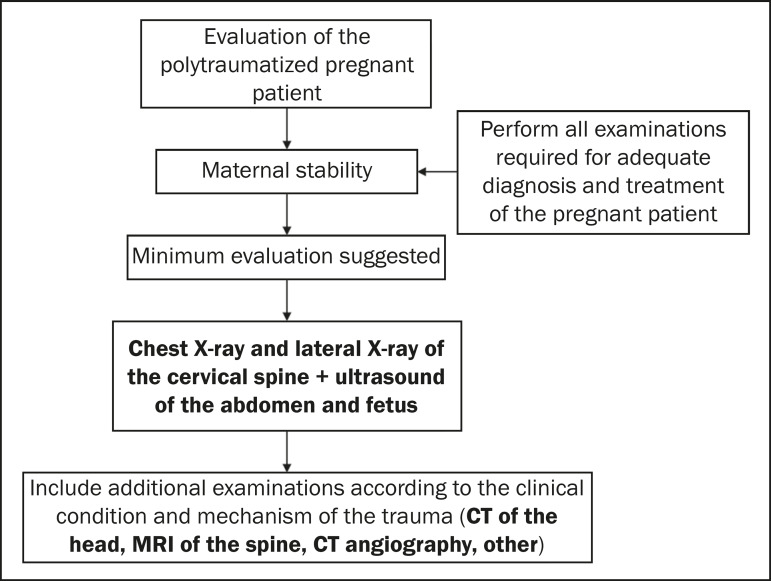
Algorithm for the imaging evaluation of polytraumatized pregnant patients.

The initial evaluation of polytraumatized pregnant patients includes radiography of the thorax/cervical spine, obstetric ultrasound, and abdominal ultrasound. Abdominal ultrasound is the imaging method of choice because it is safe to use during pregnancy, as well as being an integral part of the initial evaluation of polytraumatized patients in general. Its main purpose is to identify blood content in the abdominal cavity^(5,11)^. In cases in which these examinations are not sufficient for diagnostic clarification, additional studies should be performed, according to clinical suspicion, related to the mechanism of trauma. Additional examinations include CT of the head, thorax, abdomen, and pelvis, if required (Figure 8). When available, MRI can also play a major role in the evaluation of neurological lesions. No diagnostic or interventionist examination necessary for the definition of medical practices should be omitted or delayed, especially in the context of trauma^(5,11)^.

### Evaluation of the pregnant patient with neurological complaints

**Figure 9:**
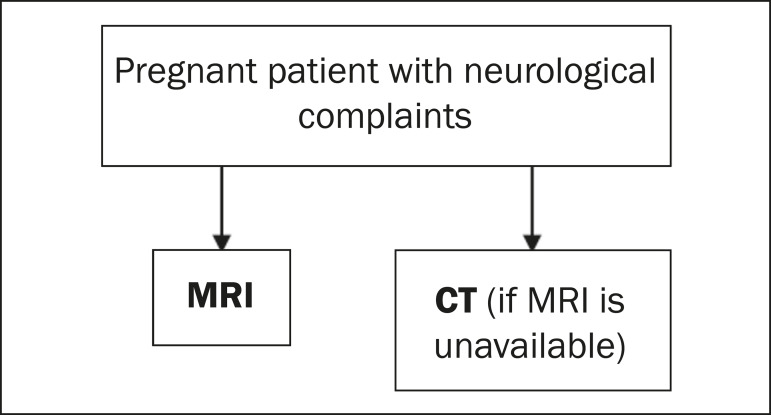
Algorithm for the imaging evaluation of neurological complaints in pregnant patients.

The main neurological conditions observed in pregnancy are headaches, venous thrombosis, preeclampsia, subarachnoid hemorrhage, posterior reversible encephalopathy syndrome, and some pituitary diseases^(21)^. The main methods for diagnosing and monitoring these conditions are CT and MRI. As previously mentioned, MRI does not involve the use of ionizing radiation and is the preferred method of investigation in the majority of neurological diseases. It should be borne in mind, however, that CT of the head, by not including the fetus in the imaging field obtained, exposes the conceptus only to low doses of radiation, and is considered relatively safe in most cases (Figure 9), provided that the principles of radiation protection are respected and that multiple successive acquisitions are not performed^(21)^.

### Evaluation of the pregnant patient with chronic disease or cancer

**Figure 10:**
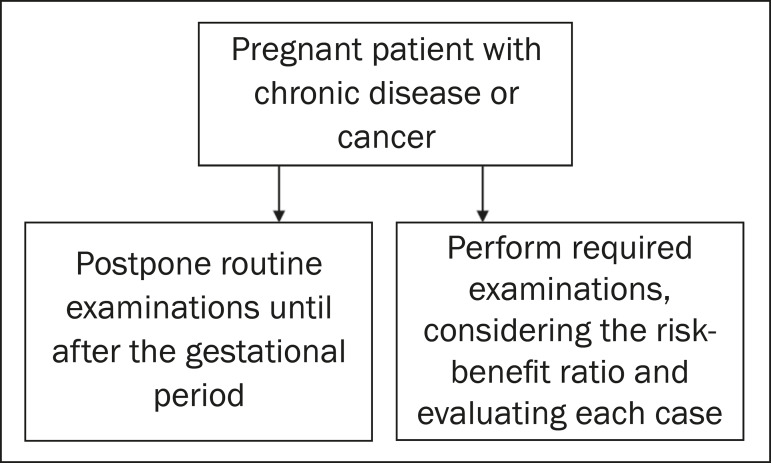
Algorithm for the imaging evaluation of chronic diseases or cancer in pregnant patients.

**Figure 11:**
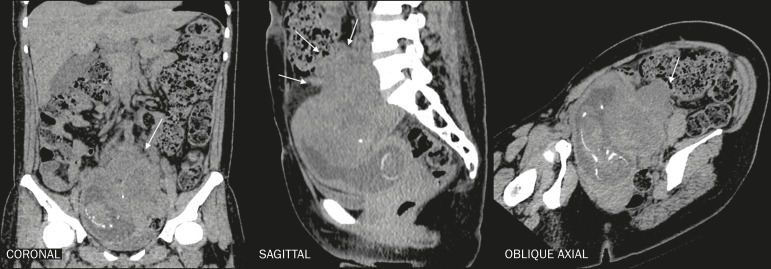
CT scan of a patient with a tumor in the organ of Zuckerkandl (arrows).

In patients with chronic diseases, such as interstitial lung disease, heart disease, and rheumatic diseases, it is recommended that the routine examinations be delayed until after pregnancy. However, if there is exacerbation of any related condition, such as unexplained respiratory worsening in patients with heart disease, or suspected opportunistic infections in immunocompromised patients, those examinations should be performed, the risk-benefit ratio being considered in each case. If the examination is considered important to define the treatment, it should not be delayed, and the diagnostic modality should be chosen on the basis of the clinical suspicion^(2,4)^. It is noteworthy that, when possible, priority should be given to methods that do not employ ionizing radiation, such as ultrasound and MRI^(3)^. In the case of cancer patients, the same principles apply, the choice of examinations being based on the underlying disease and the proposed treatment, as well as on the need for local and systemic staging (Figures 10 and 11).

## FINAL CONSIDERATIONS

Ultrasound and MRI are the preferred examinations during pregnancy. However, methods that use ionizing radiation can contribute to defining the diagnosis and treatment, when the benefits outweigh the potential risks to the fetus. Those risks are low if established dose-limiting protocols are respected and multiple acquisitions are not performed^(11)^.

Delaying or not performing an imaging study can be more harmful to the pregnant woman and to the fetus itself than the examination itself^(2,6)^.
